# NK cell cytotoxicity mediated by 2B4 and NTB-A is dependent on SAP acting downstream of receptor phosphorylation

**DOI:** 10.3389/fimmu.2013.00003

**Published:** 2013-01-22

**Authors:** Stephan Meinke, Carsten Watzl

**Affiliations:** ^1^Institute for Immunology, University HeidelbergHeidelberg, Germany; ^2^Center for Hematology and Regenerative Medicine, Karolinska InstitutetStockholm, Sweden; ^3^Leibniz Research Centre for Working Environment and Human Factors—IfADo, Department for ImmunologyDortmund, Germany

**Keywords:** natural killer cells, human, cell activation, cell surface molecules, cytotoxicity

## Abstract

2B4 (CD244) and NK-T-B-antigen (NTB-A, CD352) are activating receptors on human natural killer (NK) cells and belong to the family of signaling lymphocyte activation molecule (SLAM)-related receptors (SRR). Engagement of these receptors leads to phosphorylation of their cytoplasmic tails and recruitment of the adapter proteins SLAM-associated protein (SAP) and Ewing's sarcoma-activated transcript-2 (EAT-2). X-linked lymphoproliferative syndrome (XLP) is a severe immunodeficiency that results from mutations in the SAP gene. 2B4 and NTB-A-mediated cytotoxicity are abrogated in XLP NK cells. To elucidate the molecular basis for this defect we analyzed early signaling events in SAP knockdown cells. Similar to XLP NK cells, knockdown of SAP in primary human NK cells leads to a reduction of 2B4 and NTB-A-mediated cytotoxicity. We found that early signaling events such as raft recruitment and receptor phosphorylation are not affected by the absence of SAP, indicating the defect in the absence of SAP is downstream of these events. In addition, knockdown of EAT-2 does not impair 2B4 or NTB-A-mediated cytotoxicity. Surprisingly, EAT-2 recruitment to both receptors is abrogated in the absence of SAP, revealing a novel cooperativity between these adapters.

## Introduction

Activation of natural killer (NK) cells is regulated by the balance of signals originating from activating and inhibitory receptors (Lanier, 2008). The receptors 2B4 (CD244) and NK-T-B-antigen (NTB-A, CD352) are activating receptors on NK cells that induce cytotoxicity and cytokine production. They belong to the family of signaling lymphocyte activation molecule (SLAM)-related receptors (SRR) (Claus et al., 2008). More and more evidence is emerging demonstrating that this family of Ig-like receptors plays a role in fine-tuning of immune responses, e.g., in germinal center formation or development of innate-like T cells (Schwartzberg et al., 2009). All SRR are homophilic with the exception of 2B4 that binds to CD48, another Ig-like protein with broad expression in the hematopoietic system (Claus et al., 2008). SRR signaling is dependent on phosphorylation of their cytoplasmic domains at immunoreceptor tyrosine-based switch motifs (ITSM). In humans, phosphorylated ITSMs can recruit the small adapter proteins SLAM-associated protein (SAP) and Ewing's sarcoma-activated transcript-2 (EAT-2). These adapter molecules comprise only one Src homology 2 (SH2) domain and a short C-terminal extension (Ma et al., 2007; Claus et al., 2008). ITSM-bound SAP mediates signal transduction by recruiting the Src-family kinase FynT (Ma et al., 2007), which then phosphorylates downstream signaling molecules, e.g., Phospholipase C-γ or Vav-1 (Watzl et al., 2000; Chen et al., 2004). Signal transduction molecules recruited by ITSM-bound EAT-2 have not been identified yet.

SAP plays an important role for lymphocyte function, as mutations in SAP result in a severe immunodeficiency called X-linked lymphoproliferative syndrome (XLP). Patients suffering from XLP show impaired humoral immune responses, defects in cytotoxic lymphocytes, and often develop a fatal mononucleosis after EBV infection (Ma et al., 2007). 2B4 and NTB-A-mediated cytotoxicity are defective in NK cells from XLP patients (Ma et al., 2007). One study demonstrated that 2B4 engagement could even mediate inhibitory signals in XLP patients (Parolini et al., 2000). SAP^−/−^ mice, which display similar immune defects like XLP patients, are used as an animal model for XLP (Ma et al., 2007).

Early signaling events after binding of 2B4 to its ligand CD48 are recruitment of the receptor to lipid rafts (Watzl and Long, 2003), phosphorylation of the four ITSM by Src-family kinases (Watzl et al., 2000; Watzl and Long, 2003), association of adapter molecules SAP and EAT-2 (Tangye et al., 1999; Morra et al., 2001), and recruitment of the Src-kinase FynT (Chen et al., 2004). Similarly, engagement of NTB-A leads to phosphorylation of the two ITSM and the recruitment of SAP and EAT-2 (Bottino et al., 2001; Eissmann and Watzl, 2006).

There are contradicting reports concerning the role of SAP and SAP-recruited FynT in these events. On the one hand, there are reports suggesting that 2B4 phosphorylation takes place independently from SAP: pervanadate treatment of NK cells from XLP patients leads to 2B4 phosphorylation (Parolini et al., 2000), and antibody mediated cross-linking of 2B4 has recently been reported to induce receptor phosphorylation in NK cells from SAP^−/−^ mice, as well as from mice lacking all SAP-family adaptors (Dong et al., 2012). In addition SAP cannot be found in association with the non-phosphorylated receptor in NK cells from healthy donors (Eissmann et al., 2005), which indicates that receptor phosphorylation precedes SAP-mediated FynT recruitment. On the other hand, there are reports suggesting that SAP-mediated Fyn recruitment is necessary for 2B4 phosphorylation: one study used a transfected cell line, in which co-transfection with SAP, but not co-transfection with a non-FynT-binding mutant form allowed 2B4 phosphorylation after receptor cross-link (Chen et al., 2004). Another study analyzing NK cells from SAP^−/−^ mice found impaired 2B4 phosphorylation after receptor cross-link (Bloch-Queyrat et al., 2005). Furthermore, 2B4 has been shown to be a target of FynT-mediated phosphorylation (Eissmann et al., 2005).

In this study we wanted to investigate how SAP and EAT-2 contribute to the early signaling events after 2B4 and NTB-A engagement in order to gain further insight into the molecular basis for the defective signaling of 2B4 and NTB-A in XLP NK cells.

## Results

### Knockdown of SAP leads to reduced 2B4 or NTB-a-mediated cytotoxicity

**Figure 1:**
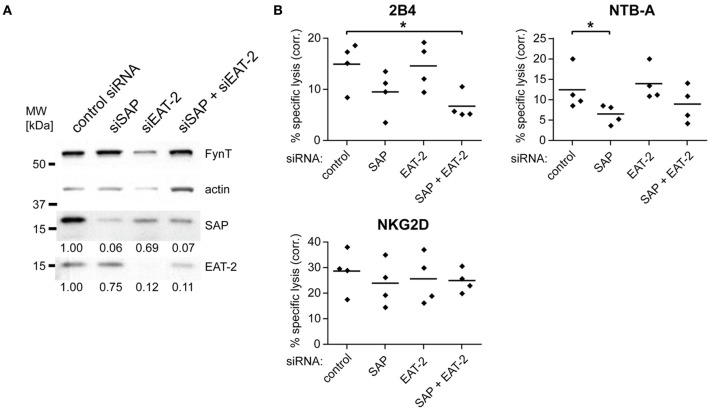
**2B4 and NTB-A-mediated cytotoxicity is dependent on the expression of SAP, but not EAT-2 in primary human NK cells.** IL-2-activated primary human NK cells were transfected with control siRNA, siRNA against SAP, against EAT-2 or both. **(A)** The knockdown was confirmed 48 h later by Western blotting of whole cell lysates. The numbers below the lanes indicate relative signal intensity. The values were normalized to the respective actin signal and the value for the control siRNA sample was set to 1. **(B)** The functional consequence of the decreased expression of the adapter molecules was tested in a 4 h ^51^Cr-release assay against P815 cells in the presence of control Ab, or antibodies against 2B4, NTB-A, or NKG2D. Results shown are from four independent experiments. Plotted is the specific lysis at an E/T ratio of 5/1 corrected by subtracting the lysis observed in the presence of control Ab for each experiment. The bars represent the mean value. Statistical significance of the reduced lysis compared to control siRNA-transfected cells was calculated using One-Way ANOVA and Dunnett's post test (^*^indicates *p* < 0.05).

To study the mechanisms underlying the reduced 2B4 or NTB-A-mediated cytotoxicity observed in NK cells from XLP patients we investigated the effect of SAP or EAT-2 knockdown in primary human NK cells. Transfection of *in vitro* propagated, IL-2-activated NK cells with siRNAs against SAP, EAT-2, or both mRNAs led to a strong reduction in protein expression of about 90% (Figure 1A). The expression level of the Src-family kinase FynT was not changed. Knockdown of SAP diminished 2B4- and NTB-A-mediated cytotoxicity (Figure 1B) similar to the defect that has been reported for NK cells from XLP patients (Ma et al., 2007). In contrast, knockdown of EAT-2 did not decrease cytotoxicity mediated by these two receptors. Furthermore, NK cells with a double knockdown showed no significant further reduction of cytotoxicity compared to NK cells with knockdown of SAP alone (Figure 1B). The observed effects were not due to general impairment of NK cytotoxicity by the knockdown, as cell lysis triggered by NKG2D engagement was similar for all transfected cells (Figure 1B). These findings confirm that the impaired cytotoxicity of XLP NK cells is caused by defective signaling after target cell contact and not due to defective NK cell development caused by the absence of SAP. The related EAT-2, which can mediate signaling pathways leading to cytotoxicity in association with the SRR CRACC (CD2-like receptor activating cytotoxic cells) (Bouchon et al., 2001), seems to regulate different pathways in 2B4 and NTB-A signaling, as its knockdown had no impact on cytotoxicity.

**Figure 2:**
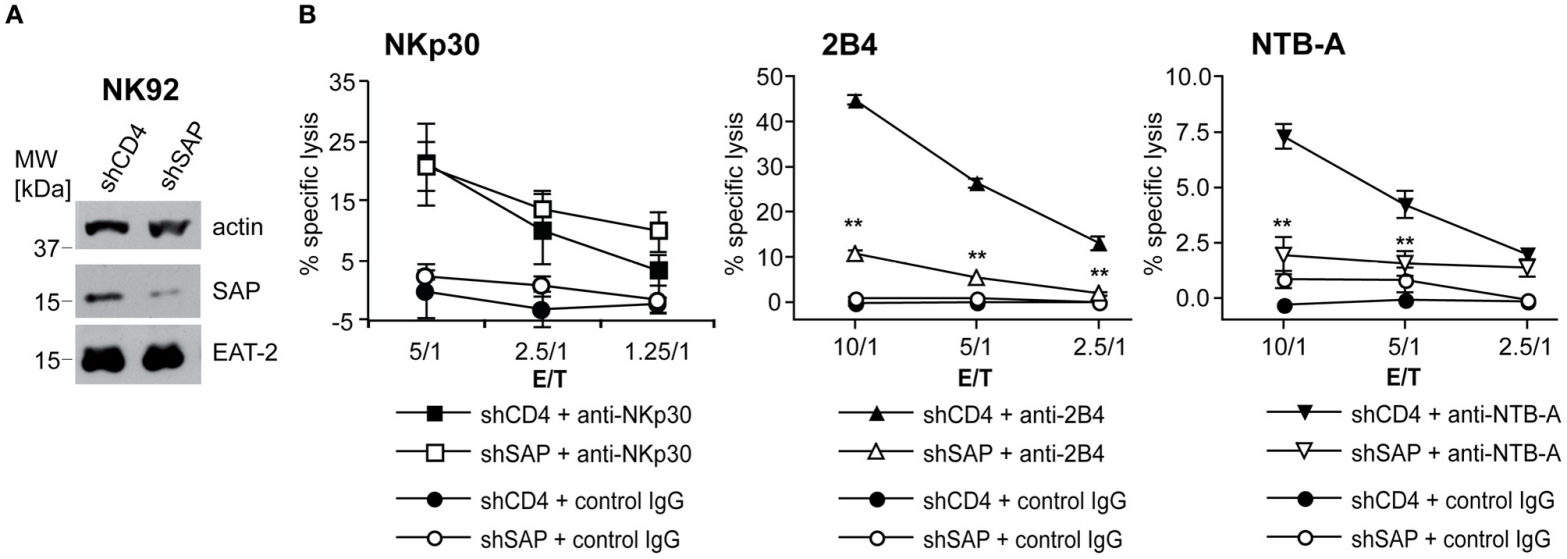
**SAP knockdown leads to diminished 2B4 or NTB-A-mediated cytotoxicity in cell lines.** To study the impact of a SAP-knockdown on NK cell cytotoxicity, NK92-C1 cells stably expressing a small hairpin RNA against SAP (shSAP) or CD4 (shCD4) mRNA (as negative control) were analyzed. **(A)** Western blot analysis of SAP and EAT-2 expression in NK92-C1 shCD4 and shSAP cells. **(B)** NK92-C1 shCD4 and shSAP were used as effector cells in a redirected ^51^Cr-release assay against P815 cells in the presence of IgG control Ab or antibodies against the receptors NKp30, 2B4, or NTB-A at different E/T ratios. Data is shown as mean ± SD of triplicates. Statistical significance of the reduced lysis by NK92-C1 shSAP compared to shCD4 cells was calculated using One-Way ANOVA and Dunnett's post test (^**^indicates *p* < 0.0001). The results are from one representative experiment out of four.

Because the numbers of primary NK cells recovered after transfection were too small for sufficient biochemical analysis of signaling events, we generated a stable knockdown of SAP by shRNA expression in the NK cell line NK92-C1. The SAP expression level in knockdown cells was about 10% of the amount expressed in control cells (Figure 2A). We also used two EAT-2 knockdown vectors, but the reduction of EAT-2 expression was only minor (data not shown). Similar to primary NK cells, NK92-C1 cells with SAP knockdown showed strongly diminished 2B4-mediated cell lysis. The already low cytotoxicity induced by NTB-A engagement in control cells was further reduced in SAP knockdown cells (Figure 2B). Like in primary cells cytotoxicity mediated by a SAP-independent receptor, NKp30, was not affected (Figure 2B). A similar, albeit weaker effect of SAP knockdown was observed in the NK-like cell line YTS (data not shown). As the knockdown had the same effect on cytotoxicity in the cell lines and the primary NK cells, we used the knockdown cell lines to analyze the molecular basis of this signaling defect.

### Recruitment of 2B4 to lipid rafts is independent of SAP

**Figure 3:**
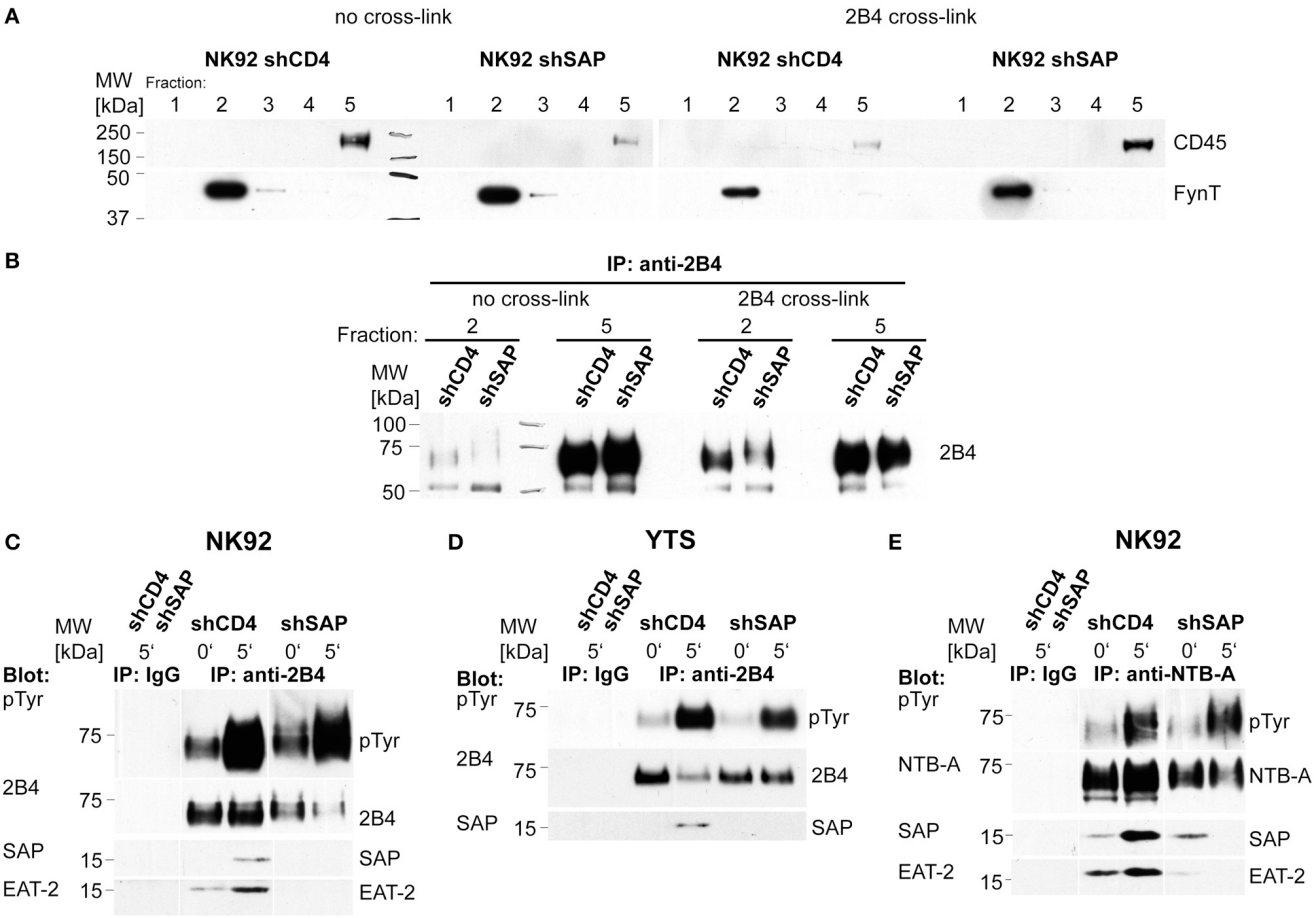
**Raft recruitment of 2B4 to lipid rafts is independent of SAP**. NK92-C1 shCD4 and shSAP were stimulated by antibody-mediated 2B4 cross-linking. Cells were lysed and lipid rafts were isolated by sucrose density gradient centrifugation. **(A)** Gradient fractions were analyzed in Western blotting. FynT served as a marker for lipid rafts, while CD45 was used to identify the soluble fraction. **(B)** 2B4 was immunoprecipitated from the soluble fraction and the fraction containing lipid rafts. Immunoprecipitates were analyzed in Western blots for the presence of 2B4. **(C)** For analysis of receptor phosphorylation NK92-C1 cell lines were mixed with equal numbers of 721.221 cells and lysed at the indicated time-points. After control immunoprecipitation with an unspecific Ab (IP: IgG) 2B4 was immunoprecipitated from the lysates. The immunoprecipitates were analyzed by Western blotting using anti-phospho-tyrosine and anti-2B4 antibodies (upper panels). Antibodies against SAP and EAT-2 were used to detect co-precipitated molecules on the same membrane (lower panels). **(D)** YTS shCD4 and shSAP were analyzed as described in **(C)**. As these cells express less EAT-2 than NK92-C1 cells we were not able to detect this adapter molecule associated with 2B4. **(E)** After mixing NK92-C1 with 721.221 cells, phosphorylation of NTB-A was analyzed in the same way as 2B4 in **(C)**. The blots shown are representative of at least three independent experiments.

One of the first events in 2B4 signaling is recruitment of the receptor to lipid rafts (Watzl and Long, 2003). Therefore, we investigated whether this early signaling event is impaired in the absence of SAP. Lipid rafts were isolated from NK92-C1 cells with stable SAP knockdown after cross-linking of 2B4 with antibodies. Western blot analysis of 2B4 immunoprecipitated from rafts and soluble fractions revealed no difference in stimulation-dependent raft recruitment of 2B4 between knockdown and control cells (Figures 3A,B). This finding indicates that SAP is not necessary for the raft recruitment of 2B4.

### Phosphorylation of 2B4 and NTB-A is independent of SAP

After recruitment to lipid rafts the ITSMs of 2B4 become phosphorylated (Watzl and Long, 2003). To analyze 2B4 phosphorylation in SAP knockdown cell lines, cells were stimulated by mixing with target cells expressing CD48. We found no difference in the induction of 2B4 phosphorylation between knockdown and control cells in NK92-C1 and YTS (Figures 3C,D). We also analyzed phosphorylation of NTB-A in NK92-C1 cells (Figure 3E). After stimulation with target cells expressing NTB-A we observed a comparable increase of NTB-A phosphorylation in SAP knockdown and control cells. These findings demonstrate that SAP is not necessary for the induction of 2B4 and NTB-A phosphorylation.

The immunoprecipitates were also analyzed for co-precipitated adapter molecules. While stimulation of 2B4 led to recruitment of SAP in control cells, we detected no stimulation-dependent association of SAP with 2B4 in SAP knockdown cells (Figures 3C,D), although the knockdown cells still had residual amounts of SAP (Figure 2 and data not shown).

The amount of 2B4-bound EAT-2 increased with elevated phosphorylation after stimulation in control NK92-C1 cells (Figure 3C). However, in cells with reduced SAP expression EAT-2 could not be detected in the immunoprecipitates despite its unchanged expression (Figure 2A).

In non-stimulated NK92-C1 cells SAP and EAT-2 could be found in association with NTB-A (Figure 3E). However, while engagement of NTB-A increased the recruitment of both adapter proteins, we could neither detect SAP nor EAT-2 in immunoprecipitates from stimulated SAP knockdown cells (Figure 3E). These results reveal an unknown cooperativity between these adapters and indicate that SAP facilitates the binding of EAT-2 to phosphorylated 2B4 and NTB-A.

## Discussion

In this study we used RNAi-mediated knockdown of SAP to investigate the molecular basis of the NK cell defect in XLP and to elucidate the role of this adapter molecule in the early events of SRR signaling. Our results show that the removal of functional SAP in *in vitro* propagated, IL-2 activated NK cell, or human NK cell lines is sufficient to generate the XLP phenotype.

While knockdown of SAP impaired cytotoxicity triggered by 2B4 and NTB-A, we could not see any difference in the early signaling events of these two receptors, i.e., raft recruitment and receptor phosphorylation. This suggests that the impairment of signaling is due to defective recruitment of FynT to phosphorylated receptors, which is essential to mediate down-stream signaling events (Bloch-Queyrat et al., 2005). Our finding is in contrast to a report of absent 2B4 phosphorylation in a study on NK cells from SAP^−/−^ mice (Bloch-Queyrat et al., 2005). While we cannot exclude an off-target effect of our RNAi approach which could contribute to this difference, a recent study using murine NK cells came to the same conclusion as our study: Dong et al. found similar levels of phosphorylated 2B4 after receptor cross-linking in NK cells from SAP^−/−^ and wild-type mice, as well as in mice transgenic for the SAP mutant R78A that cannot recruit FynT (Dong et al., 2012).

Our data also demonstrate that SAP is necessary for the binding of EAT-2 to 2B4 and NTB-A. This novel cooperativity between these adapters suggests that the absence of SAP will also disrupt the unknown signaling pathways induced by EAT-2. These pathways do not seem to be necessary for a cytotoxic response, as we did not find a reduction of 2B4 or NTB-A-mediated cytotoxicity after EAT-2 knockdown. This is in contrast to observations made with mouse NK cells. Cytotoxicity in NK cells from EAT-2^−/−^ mice has been reported to be either reduced (Wang et al., 2010) or enhanced (Roncagalli et al., 2005) depending on the strain tested. This could be due to the difference between species or to the difference in experimental approach. It could be possible that EAT-2 plays a role during NK cell development that has an impact on cytotoxicity. In this case NK cells from a knockout animal would show a difference, while a knockdown in mature cells shortly before cytotoxicity is tested would not affect the response. To answer this question a deeper knowledge of the EAT-2-mediated signaling pathways will be necessary.

## Materials and methods

### Antibodies and reagents

The following antibodies were used in this study: IgG1 control Ab MOPC21 (Sigma-Aldrich), anti-2B4 clone C1.7 (Immunotech), anti-NKG2D (R&D Systems), anti-CD45 clone 69/CD45, and anti-Fyn clone 25 (both BD Transduction Laboratories), anti-phospho-tyrosine clone 4G10 (upstate), polyclonal rabbit anti-actin Ab (Sigma-Aldrich). The mouse monoclonal antibodies anti-NTB-A clone NT-7, anti-SAP clone SAP 23.1.5, anti-NKp30 clone p30–15, and the rabbit polyclonal antibodies anti-2B4, anti-NTB-A, and anti-EAT-2 have been generated in our lab as described previously (Watzl and Long, 2003; Flaig et al., 2004; Eissmann et al., 2005; Eissmann and Watzl, 2006; Byrd et al., 2007). The goat-anti-mouse-IgG Ab used for cross-linking was from Jackson ImmunoResearch Laboratories.

### Cells

Peripheral blood mononuclear cells (PBMC) were isolated from buffy coats or whole blood using density centrifugation over lymphocyte separation medium (PAA, Pasching, Germany). Polyclonal NK cells were purified from PBMC by negative selection using a NK cell negative isolation kit (Invitrogen). NK cells were between 90% and 99% NKp46^+^, CD3^−^, and CD56^+^. Cells were resuspended in IMDM with 10% human serum (PromoCell, Heidelberg, Germany), penicillin/streptomycin containing 200 IU/mL IL-2 (National Institutes of Health cytokine repository), 1 μg/mL PHA-P and 5 ng/mL recombinant human IL-15 (R&D Systems), mixed with irradiated JY cells (5 × 10^5^ cells/mL), and plated in 96-well round bottom plates.

The following cell lines were used in this study: the EBV-transformed, human B cell line 721.221, the murine mastocytoma line P815 (both grown in IMDM, 10% FCS, penicillin/streptomycin), cells of the murine pre-B cell line BA/F3 stably transduced with the retroviral vector pBABE containing GFP, human CD48, or human NTB-A Y123F (Eissmann and Watzl, 2006) (all grown in RPMI 1640, 10% FCS, 50 μM 2-mercaptoethanol, penicillin/streptomycin, and selected with puromycin), stable CD4 or SAP knockdown lines generated from the cell lines NK92-C1 (grown in Alpha MEM, 12.5% FCS, 12.5% donor horse serum, 50 μM 2-mercaptoethanol, and penicillin/streptomycin), and YTS (grown in IMDM, 12.5% FCS, 50 μM 2-mercaptoethanol, and penicillin/streptomycin) by transduction with the retroviral vector pSHAG-MAGIC2 (Biocat) (Eissmann and Watzl, 2006).

### Transfection of primary human NK cells

Primary NK cells were transfected after 1 week of culture using the nucleofection method (Lonza). The following siRNAs were used: non-targeting siRNA #1 (Thermo Scientific), Hs_SH2D1A_3 for SAP knockdown, and Hs_SH2D1B_1 for EAT-2 knockdown (both from Qiagen). 2–3 × 10^6^ cells per sample were transfected with 1 pmol of siRNA using the Nucleofector solution for human macrophages and the nucleofection program X-001, following the manufacturer's instructions at all other steps.

### Cytotoxicity assays, antibody cross-linking, cell mixing, and raft isolation

Cytotoxicity ^51^Cr release assays, antibody-mediated cross-linking, raft isolation, cell mixing, and western blot analysis were performed as described previously (Watzl and Long, 2003; Eissmann and Watzl, 2006). For immunoprecipitation of 2B4 from raft fractions 0.5 mL from the respective fractions were diluted with an equal volume of lysis buffer containing 0.5% Triton X-100 and incubated for 1 h at 4°C with 0.8 μg C1.7 Ab coupled to Dynabeads protein G (Invitrogen). The beads were then treated as described for the immunoprecipitation from cell lysates (Eissmann and Watzl, 2006). Western blotting signals were quantified using a densitometer (GS800, BioRad).

### Statistical analysis

Statistical analysis was performed using Prism 4.0 (GraphPad Software Inc.).

#### Conflict of interest statement

The authors declare that the research was conducted in the absence of any commercial or financial relationships that could be construed as a potential conflict of interest.
